# Development of the GDF-TRACK-AKI Score for Predicting Acute Kidney Injury in Patients with Rhabdomyolysis Due to Excessive Exercise or Trauma

**DOI:** 10.3390/medicina61071116

**Published:** 2025-06-20

**Authors:** Oğuzhan Zengin, Burak Göre, Melike Yakut, Mustafa Yaylalı, Muhammet Göv, Safa Dönmez, Gülhan Kurtoğlu Çelik, Gül Pamukçu Günaydın, Esma Andaç Uzdoğan, Emra Asfuroğlu Kalkan, İhsan Ateş

**Affiliations:** 1 Department of Internal Medicine, University of Health Sciences, Ankara Bilkent City Hospital, Ankara 06800, Türkiye; emra.kalkan@hotmail.com (E.A.K.); dr.ihsanates@hotmail.com (İ.A.); 2 Department of Internal Medicine, Çerkeş State Hospital, Çankırı 18600, Türkiye; 3 Department of Internal Medicine, Çifteler State Hospital, Eskişehir 26700, Türkiye; melikeyakut95@gmail.com; 4 Department of Internal Medicine, Ankara Bilkent City Hospital, Ankara 06800, Türkiye; myaylal937@gmail.com; 5 Department of Internal Medicine, Mihalıççık Gün Sazak State Hospital, Eskişehir 26900, Türkiye; govmmmt@gmail.com; 6 Emergency Medicine Department, Ankara Bilkent City Hospital, Ankara 06800, Türkiye; drsafa0131@gmail.com (S.D.); kurtoglugulhan@yahoo.com (G.K.Ç.); gulpamukcu@gmail.com (G.P.G.); 7 Department of Medical Biochemistry, Ankara Bilkent City Hospital, Ankara 06800, Türkiye; auzdogan@gmail.com

**Keywords:** rhabdomyolysis, acute kidney injury, growth differentiation factor-15, creatine kinase, GDF-TRACK-AKI score, trauma, excessive exercise

## Abstract

*Background and Objectives*: Rhabdomyolysis is a disorder in which skeletal muscle tissues are damaged, resulting in the escape of their internal substances into the blood circulation. Acute kidney injury (AKI) is a serious complication of rhabdomyolysis that necessitates early recognition to ensure effective clinical management. The objective of this research was to create a practical scoring tool for forecasting AKI in patients experiencing rhabdomyolysis due to trauma or excessive exercise. *Materials and Methods*: A novel scoring system, termed the growth differentiation factor-15-trauma-creatine kinase acute kidney injury score (GDF-TRACK-AKI score), was established. The model integrates serum levels of growth differentiation factor-15 (GDF-15), creatine kinase (CK), and occurrence of rhabdomyolysis associated with trauma. Clinical and biochemical data were prospectively collected, and the model’s predictive performance was evaluated using receiver operating characteristic ROC curve analysis. *Results*: Among patients with rhabdomyolysis, those who developed AKI had significantly higher GDF-TRACK-AKI scores (median: 3.00 (IQR: 2.00)) compared to patients without AKI (median: 0.48 (IQR 0.89); *p* < 0.001). Serum CK and GDF-15 levels were also markedly elevated in the AKI group (*p* < 0.001). ROC analysis identified a cut-off value of 2.5, providing 67% sensitivity and 98% specificity. Patients with scores ≥ 2 demonstrated a significantly increased risk of AKI. *Conclusions*: Designed as a practical and dependable tool, the GDF-TRACK-AKI score facilitates prompt identification of kidney injury in patients whose rhabdomyolysis is linked to either trauma or vigorous activity. The integration of trauma history with GDF-15 and CK biomarker data improves risk stratification precision and supports timely treatment decisions. To verify its practical utility and prognostic capabilities, the GDF-TRACK-AKI score should undergo additional evaluation across expansive and demographically varied clinical populations.

## 1. Introduction

Rhabdomyolysis refers to the deterioration of skeletal muscle tissue caused by diverse factors, which leads to the escape of internal cellular substances into the bloodstream [1]. Disruption of the muscle cell membrane enables compounds such as myoglobin, creatine kinase (CK), lactate dehydrogenase (LDH), phosphate, and potassium to enter the circulatory pathways [2]. AKI is a major and dangerous complication, largely caused by myoglobin’s harmful effects on the kidney’s tubular cells [3]. Rhabdomyolysis is commonly linked to several key causes, including physical trauma, vigorous exercise, seizures, elevated body temperature, mitochondrial metabolic diseases, specific drugs, toxins, and infections [4].

The primary mechanism involves impaired ATP synthesis that alters ionic balance, triggering an abnormal calcium influx into the cell. This process stimulates lysosomal enzymes, resulting in self-digestion of the cell [5]. Continuous muscle injury releases myoglobin, which significantly harms and kills renal tubular tissue, especially when urine acidity is elevated [6].

Rhabdomyolysis manifests with a wide spectrum of symptoms, largely influenced by the underlying etiology and severity, from minor fatigue to potentially life-threatening complications. In mild cases, patients may report only muscle aches, fatigue, or a general sense of malaise. However, in more severe instances, symptoms may include pronounced muscle weakness, flank pain, decreased urine output, nausea, vomiting, tachycardia, respiratory distress, and, in some cases, neurological signs. Existing literature indicates that a significant proportion of patients exhibit not only primary complaints such as nausea, vomiting, and oliguria, but also signs of fluid overload, hypertension, muscle cramps, flank discomfort, and burning sensations in the extremities. This clinical variability highlights the importance of maintaining a high index of suspicion and adopting a vigilant diagnostic approach, particularly in individuals with predisposing risk factors, to facilitate early recognition and timely intervention [7,8,9,10].

Although the biochemical cascade underlying muscle breakdown is similar, trauma-induced or excessive exercise-induced rhabdomyolysis differs clinically and prognostically [11]. Trauma-related cases are commonly associated with crush injuries, prolonged compression, or major surgical interventions, often leading to concurrent soft tissue and vascular damage. This amplifies myoglobin-induced nephrotoxicity through mechanisms such as circulatory compromise, local inflammation, and fluid depletion [12]. Consequently, AKI risk is substantially higher in trauma-induced rhabdomyolysis [13]. In contrast, excessive exercise-induced rhabdomyolysis typically occurs in otherwise healthy individuals following unaccustomed intense exertion, with a relatively lower risk of complications unless exacerbated by dehydration, extreme environmental conditions, or underlying metabolic disorders [14]. Correct identification of the underlying etiology is thus crucial for tailoring management and preventing complications.

While serum CK levels are routinely monitored as an indicator of muscle injury in rhabdomyolysis, several studies have shown that CK alone does not reliably predict AKI development [15]. There can be discordance between CK levels and renal outcomes; some patients exhibit remarkably high CK levels without kidney dysfunction, whereas others develop AKI despite relatively lower CK elevations [16]. This suggests that multiple systemic factors, including fluid status, acid-base balance, and individual susceptibility, modulate the risk of nephrotoxicity [17]. Therefore, a multidimensional approach considering various clinical and biochemical parameters is likely to yield better predictive accuracy [18].

GDF-15 is a stress-responsive cytokine belonging to the transforming growth factor-beta (TGF-β) superfamily. Under physiological conditions, it is expressed at low levels in most tissues; however, its expression is markedly upregulated in response to cellular stress, inflammation, hypoxia, and tissue injury. GDF-15 is secreted by various cell types, including renal tubular epithelial cells, cardiomyocytes, macrophages, and adipocytes, particularly under metabolic or oxidative stress conditions. In renal physiology, GDF-15 plays a role in modulating inflammation, preventing fibrosis, and supporting cellular survival mechanisms. Experimental studies have demonstrated that GDF-15 provides renoprotective effects in models of ischemia-reperfusion injury by regulating immune responses and mitigating tubular damage. Clinically, elevated GDF-15 levels in serum and urine have been associated with unfavorable outcomes and pathological findings in both acute and chronic kidney diseases. Furthermore, GDF-15 has been shown to rise earlier than traditional renal biomarkers, highlighting its potential utility as an early indicator of AKI. However, it is important to note that GDF-15 is not kidney-specific; its expression may also be influenced by systemic conditions such as cardiovascular disease, malignancies, and chronic inflammatory states [19,20,21,22,23].

In this study, we aimed to develop a novel predictive model for the occurrence of AKI in cases of trauma-induced or excessive exercise-induced rhabdomyolysis. Previous research has demonstrated that growth GDF-15 is markedly elevated in response to cellular stress, inflammation, and tissue injury and may rise earlier than traditional renal biomarkers in settings such as ischemic kidney injury. Based on these findings, GDF-15 has been proposed as a potential early biomarker for AKI. We hypothesized that combining GDF-15 levels with CK measurements and trauma etiology would enhance the predictive accuracy for AKI in patients with rhabdomyolysis. Accordingly, we developed the GDF-TRACK-AKI score to facilitate early risk stratification and clinical intervention. The study involved a comprehensive comparative analysis of three cohorts: trauma-induced rhabdomyolysis, excessive exercise-induced rhabdomyolysis, and a healthy control group.

## 2. Materials and Methods

### 2.1. Study Design and Ethical Approval

This was a single-center, prospective observational study conducted at the Departments of Emergency Medicine and Internal Medicine of Ankara Bilkent City Hospital. The study period spanned from 21 February 2024, to 21 February 2025 and included patients hospitalized with a diagnosis of rhabdomyolysis as well as healthy control participants. The study protocol was approved by the Ankara Bilkent City Hospital Ethics Committee (Approval Number: E2-24-6475 Date: 21 February 2024) and conducted in accordance with the principles of the Declaration of Helsinki. Written informed consent was obtained from all participants.

### 2.2. Study Population and Participant Selection

**Figure 1:**
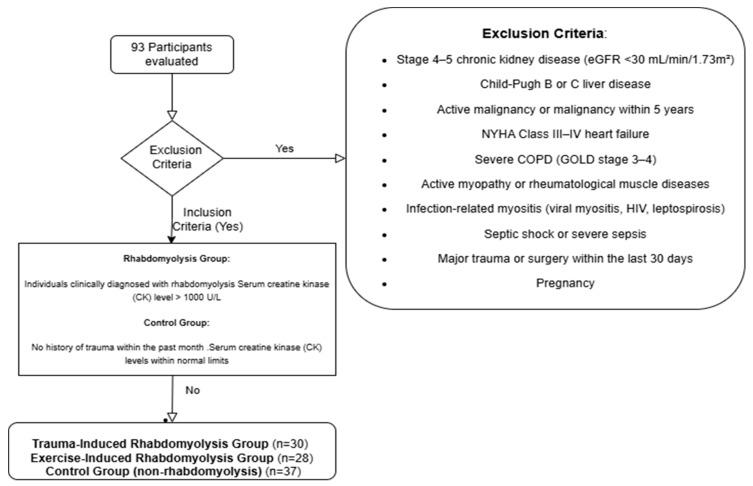
Study flowchart.

Individuals aged 18 years and older were included in the study. The rhabdomyolysis group comprised only patients diagnosed with rhabdomyolysis following either excessive exercise or trauma. In addition to clinical findings consistent with rhabdomyolysis, a serum CK level exceeding 1000 U/L was required for inclusion. The control group was composed of healthy individuals matched for age and sex, with no history or diagnosis of rhabdomyolysis. This selection strategy aimed to minimize the heterogeneity associated with rhabdomyolysis etiology and to eliminate secondary effects stemming from inflammatory, infectious, or metabolic causes of muscle and kidney injury. Consequently, the study sought to more specifically evaluate the association between GDF-15 levels and rhabdomyolysis-related muscle injury and accompanying AKI. Participants were excluded if they had Child–Pugh class B or C liver disease, as advanced cirrhosis may alter biomarker levels due to associated metabolic disturbances and hypoperfusion that affect renal function. Similarly, patients with New York Heart Association (NYHA) class III or IV heart failure were excluded, since heart failure can both predispose individuals to AKI by reducing renal perfusion and independently elevate GDF-15 levels. Individuals with stage 4 or 5 chronic kidney disease (GFR < 30 mL/min/1.73 m^2^) were also excluded due to the difficulty in distinguishing AKI related to rhabdomyolysis from underlying chronic renal dysfunction and the potential for chronically elevated biomarker levels such as GDF-15. Patients with active malignancy or a history of cancer within the past five years were excluded as well. In such cases, tumor lysis, paraneoplastic syndromes, or chemotherapy may lead to elevations in CK and GDF-15 levels, and malignancies can directly affect kidney function. Patients with severe chronic obstructive pulmonary disease (COPD), defined as GOLD stage 3–4, were also excluded, as chronic hypoxemia and systemic inflammation in this population may increase GDF-15 levels. Moreover, COPD-related muscle wasting and renal involvement could confound biomarker results. Rheumatologic diseases involving muscle (e.g., polymyositis, dermatomyositis, systemic lupus erythematosus) were excluded due to the potential for immune-mediated muscle injury to independently elevate CK and GDF-15 levels. In addition, renal involvement such as glomerulonephritis in these conditions may result in AKI unrelated to rhabdomyolysis. Infectious conditions that may involve muscle (e.g., viral myositis, HIV, leptospirosis) were also exclusionary, as inflammation-induced elevations in CK and GDF-15 could occur, and these infections can directly damage renal tissue. Patients with septic shock or severe sepsis were not included in the study, as these conditions may increase GDF-15 levels independently of rhabdomyolysis. Similarly, those who had undergone major surgery in the past 30 days were excluded due to the potential for both trauma- and inflammation-induced muscle and kidney injury. Pregnancy was also an exclusion criterion, as physiological changes during pregnancy can affect glomerular filtration rate and biomarker levels. In summary, excluding individuals with inflammatory myopathies or infection-related muscle involvement allowed for the assessment of changes in CK and GDF-15 levels specifically in the context of rhabdomyolysis due to exertion or trauma, without the confounding influence of systemic inflammation or immune-mediated muscle injury. This approach was adopted to enhance the ability of the biomarkers to reflect rhabdomyolysis-related AKI. The exclusion criteria—specifically regarding liver disease, heart failure, and COPD—were determined based on internationally accepted clinical classification systems. Detailed information regarding these systems is presented in Table 1 (Child–Pugh classification), Table 2 (NYHA classification), and Table 3 (GOLD classification) [24,25,26]. Study flowchart is shown in Figure 1.

### 2.3. Sample Size Calculation

The sample size was calculated to evaluate the predictive ability of the GDF-TRACK-AKI score for AKI development among rhabdomyolysis patients. Using G*Power 3.1 software, with an effect size of 0.8 (Cohen’s d), a significance level (α) of 0.05, and a power (1–β) of 0.80, it was determined that at least 26 participants per group were required to detect a significant difference. To account for potential data loss, the sample size was increased, and a total of 63 rhabdomyolysis patients and 37 control participants were ultimately included.

### 2.4. Data Collection

Sociodemographic data (age, sex), clinical characteristics (etiology of rhabdomyolysis, AKI development, need for renal replacement therapy (RRT), and mortality), and laboratory findings (serum CK, creatinine, urea, electrolytes, liver enzymes, bilirubin levels, LDH, white blood cell (WBC) count, hemoglobin, platelet count, and serum GDF-15 levels) were prospectively recorded using standardized forms.

All laboratory analyses were performed at the hospital’s central laboratory using automated biochemical analyzers. Serum GDF-15 levels were measured using a Feiyuebio Human GDF-15 ELISA kit (96T, Feiyuebio, Wuhan, China) according to the manufacturer’s instructions, employing a validated enzyme-linked immunosorbent assay (ELISA) method. Blood samples were processed within two hours of collection; samples that could not be immediately analyzed were stored at −80 °C until testing.

### 2.5. Clinical Definitions

AKI was defined according to the kidney disease: Improving Global Outcomes (KDIGO) 2012 criteria. AKI can be diagnosed if any one of the following is present: (1) an increase in serum creatinine of ≥0.3 mg/dL within 48 h; (2) a 1.5-fold increase from baseline, which has occurred within the prior 7 days; or (3) a urine output of <0.5 mL/kg/h for 6 h. Indications for renal replacement therapy were determined by attending nephrologists based on clinical status.

### 2.6. Development of the GDF-TRACK-AKI Score

To facilitate early prediction of AKI development in rhabdomyolysis, the GDF-TRACK-AKI score was developed based on three key parameters: serum CK levels ≥ 50,000 U/L, serum GDF-15 levels within specific thresholds (between 3500 and 5000 pg/mL and ≥5000 pg/mL), and the presence of trauma-related rhabdomyolysis. It was hypothesized that the risk of AKI would increase progressively with higher scores.

### 2.7. Statistical Analysis

Statistical analyses were conducted using IBM SPSS Statistics version 26.0 (IBM Corp., Armonk, NY, USA). Categorical variables were presented as counts (n) and percentages (%), and differences between groups were assessed using the Chi-square test or Fisher’s exact test, as appropriate. The distribution of continuous variables was assessed using the Kolmogorov–Smirnov test. Since most continuous variables were not normally distributed, they were summarized as median and interquartile range (IQR), and comparisons between groups were performed using the Mann–Whitney U test or Kruskal–Wallis test. Correlations between serum GDF-15 levels and other laboratory parameters were analyzed using Spearman’s rank correlation. The diagnostic performance of the GDF-TRACK-AKI score was evaluated using ROC curve analysis, with calculation of the area under the curve (AUC), optimal cut-off point, sensitivity, specificity, positive predictive value (PPV), negative predictive value (NPV), and odds ratios (OR). A two-sided *p*-value of <0.05 was considered statistically significant.

## 3. Results

The study enrolled a total of 93 participants, categorized into three groups: trauma-induced rhabdomyolysis (n = 30), excessive exercise-induced rhabdomyolysis (n = 28), and healthy controls without rhabdomyolysis (n = 37). Demographic and clinical characteristics were compared among these groups. As summarized in Table 4 regarding sex distribution, the trauma group included 60.0% males and 40.0% females, the control group comprised 48.7% males and 51.3% females, and the excessive exercise group consisted of 78.6% males and 21.4% females. A statistically significant difference in sex distribution was observed across the groups (*p* = 0.046), which is likely attributable to the higher incidence of trauma or excessive exercise rhabdomyolysis among males, rather than an inherent sex-related susceptibility to the condition. RRT was administered exclusively in the trauma group, with 83.33% of patients requiring RRT. No patients in the control or excessive exercise groups underwent RRT (*p* = 0.001). Mortality was observed in one patient (3.33%) from the trauma group, while no deaths were reported in the control or excessive exercise groups (*p* = 0.335). AKI occurred in 83.33% of trauma-induced rhabdomyolysis patients, compared to 7.14% in the excessive exercise group and none in the control group (*p* = 0.001), highlighting the higher renal complication risk associated with traumatic muscle injury. The age distribution also showed a significant difference between the groups (*p* = 0.001). Participants in the control group were significantly older (median 75.0, IQR 19.0 years) than those in the trauma group (median 33.0, IQR 14.25 years) and excessive exercise group (median 22.5, IQR 14.25 years). Serum GDF-15 levels were highest in the trauma group (median 5741.50 pg/mL, IQR 2830.50), followed by the excessive exercise group (median 2958.50 pg/mL, IQR 1386.00) and the control group (median 2378.00 pg/mL, IQR 1314.00). The difference was statistically significant (*p* = 0.001), suggesting that GDF-15 levels correlate with the severity and etiology of muscle injury and associated kidney dysfunction. Categorical variables were assessed through the Chi-square test, whereas continuous variables, given their non-normal distribution, were examined using the Kruskal–Wallis test. Statistical significance was defined as a *p*-value < 0.05.

As summarized in Table 5, correlation analysis revealed several statistically significant associations between serum GDF-15 levels and various laboratory parameters. GDF-15 exhibited moderate positive correlations with creatine kinase (r = 0.517, *p* = 0.001), creatinine (r = 0.517, *p* = 0.001), urea (r = 0.649, *p* = 0.001), alkaline phosphatase (r = 0.281, *p* = 0.006), total bilirubin (r = 0.270, *p* = 0.008), potassium (r = 0.228, *p* = 0.026), calcium (r = 0.207, *p* = 0.044), and WBC (r = 0.597, *p* = 0.001). In contrast, significant negative correlations were observed between GDF-15 and glomerular filtration rate (r = −0.568, *p* = 0.001), as well as serum sodium levels (r = −0.409, *p* = 0.001). These findings suggest that higher GDF-15 concentrations are associated with impaired renal function, elevated metabolic stress, and systemic inflammatory markers. No statistically significant correlations were found between GDF-15 and uric acid, aspartate aminotransferase, alanine aminotransferase, gamma-glutamyl transferase, LDH, direct bilirubin, hemoglobin, or platelet count (*p* > 0.05 for all). Notably, the reported correlation coefficient for hemoglobin (r = 0.008, *p* = 0.804) appears inconsistent and may indicate a data entry or analysis anomaly that warrants verification. Collectively, these results underscore the relationship between GDF-15 and biochemical indicators of renal dysfunction, hepatic enzyme activity, and systemic inflammation.

The comparison of serum GDF-15 levels across different clinical categories is summarized in Table 6. No statistically significant difference was found between female (median: 3267.00 pg/mL, IQR: 2222.00) and male participants (median: 2991.50 pg/mL, IQR: 2471.00; *p* = 0.596). However, GDF-15 levels were markedly elevated in patients who received renal replacement therapy (median: 5794.00 pg/mL, IQR: 2125.50) compared to those who did not (median: 2639.00 pg/mL, IQR: 1276.75; *p* = 0.001). Similarly, patients with AKI exhibited significantly higher GDF-15 levels (median: 5756.00 pg/mL, IQR: 2006.00) than those without AKI (median: 2608.50 pg/mL, IQR: 1251.75; *p* = 0.001). When categorized by the etiology of rhabdomyolysis, GDF-15 levels were significantly higher in the trauma group (median: 5741.50 pg/mL, IQR: 2830.50) than in the excessive exercise group (median: 2958.50 pg/mL, IQR: 1386.00; *p* = 0.001). Moreover, individuals diagnosed with rhabdomyolysis had significantly higher GDF-15 levels (median: 3580.50 pg/mL, IQR: 3108.50) compared to healthy controls (median: 2378.00 pg/mL, IQR: 1314.00; *p* = 0.001). These findings indicate that GDF-15 levels are strongly associated with renal injury severity and the underlying cause of muscle damage.

Table 7 presents the comparison of laboratory findings between patients with trauma or excessive exercise-induced rhabdomyolysis with and without AKI. Patients with AKI had significantly higher GDF-TRACK-AKI scores (median: 3.00, IQR: 2.00) compared to those without AKI (median: 0.00, IQR: 0.00; *p* = 0.001). Similarly, levels of creatine kinase, creatinine, urea, and LDH were significantly elevated in the AKI group, while the glomerular filtration rate was markedly reduced (all *p* < 0.001). Liver enzyme levels showed mixed patterns: aspartate aminotransferase was significantly lower in the AKI group (*p* = 0.001), while alanine aminotransferase was moderately higher (*p* = 0.044). Significant electrolyte imbalance was also observed in terms of elevated potassium levels in the AKI group (*p* = 0.001), whereas sodium and calcium differences did not reach statistical significance. Hemoglobin and platelet counts were significantly lower in the AKI group, while WBC was higher, reflecting a more severe systemic response. These findings highlight distinct biochemical and hematological profiles associated with AKI in this specific subgroup of rhabdomyolysis patients and underscore the diagnostic value of the GDF-TRACK-AKI score.

**Figure 2:**
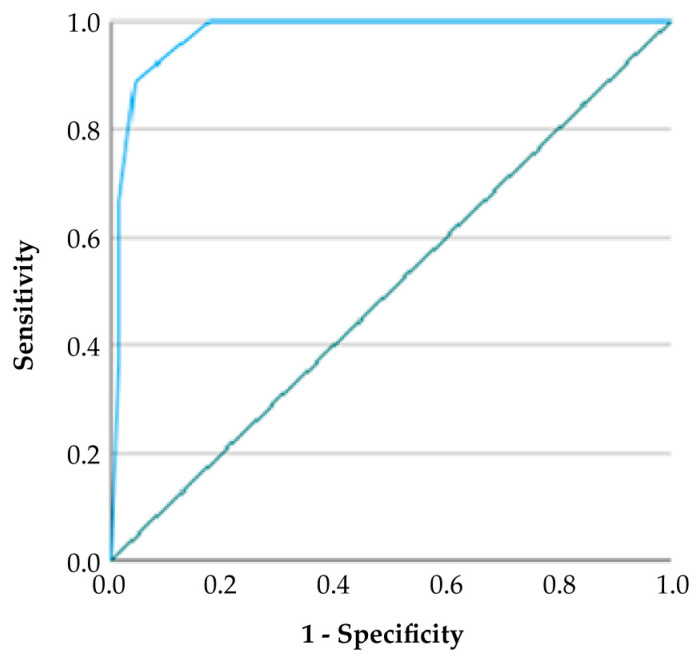
Receiver operating characteristic (ROC) curve analysis of GDF-TRACK-AKI scores for predicting acute kidney injury. The area under the curve (AUC) was 0.918 (95% CI: 0.857–0.979), indicating excellent diagnostic performance.

The GDF-TRACK-AKI score differed significantly based on the presence of AKI in patients with trauma or excessive exercise-induced rhabdomyolysis. Patients with AKI had a markedly higher median score of 3.00 (IQR: 2.00), while those without AKI had a median score of 0.00 (IQR: 0.00), indicating a statistically significant difference (*p* = 0.001). These findings suggest that the GDF-TRACK-AKI score is a robust marker for predicting AKI in this patient population. As shown in Figure 2, boxplot visualization highlights the distinct separation between groups, with scores clustering at higher values in the AKI group and remaining low in the non-AKI group. Distribution of GDF-Track AKI scores according to AKI status is shown in Table 8.

According to ROC curve analysis, the GDF-TRACK-AKI score showed high predictive accuracy for AKI, as reflected by an AUC value of 0.918 and a 95% confidence interval between 0.857 and 0.979. At the optimal cut-off value of 2.5, the score demonstrated a sensitivity of 67.0% and a specificity of 98.0%, yielding a positive predictive value (PPV) of 94.4% and a negative predictive value (NPV) of 81.4%. The Youden Index was calculated as 0.65, indicating a strong balance between sensitivity and specificity (Table 9).

## 4. Discussion

In this study, we created a scoring system that can predict the risk of developing AKI in patients with rhabdomyolysis. The basic parameters used in creating the scoring system are CK levels above 50,000 U/L, GDF-15 levels within the specified threshold values (between 3500 and 5000 pg/mL and above 5000 pg/mL), and whether rhabdomyolysis developed due to trauma.

A scoring system for early prediction of AKI due to rhabdomyolysis has not yet been clearly developed [27]. The last several years have witnessed a rise in rhabdomyolysis cases, especially with the increase in excessive exercise, traffic accidents, and traumas [28]. In addition, the number and severity of natural disasters are increasing, which increases the incidence of rhabdomyolysis and related renal failure due to crush injuries. In a study conducted after the Kahramanmaraş earthquake in 2023, the mortality rate in patients who developed crush-related AKI was reported as 12.5% [29]. This finding shows that rhabdomyolysis is not only a condition that affects muscle tissue but can also affect vital organs, leading to serious complications and mortality [30]. Therefore, there is a need for a practical and reliable scoring system that evaluates different clinical and laboratory data together, which will enable early diagnosis and intervention. In the literature, it has been observed that age, statin use, high creatinine and lactate levels, and initial serum creatine phosphokinase (CPK) level are significant in predicting the development of AKI. In addition, the course of change in CPK levels over time also stands out as an important marker in predicting the clinical outcomes observed in patients diagnosed with rhabdomyolysis [31]. However, there is still no objective method that can predict the risk of AKI in these patients. This situation often makes it difficult for patients to reach comprehensive health centers at the right time [3]. Our scoring system aims to ease the difficulties in clinical follow-up of rhabdomyolysis. With this method, it will be possible to evaluate at an early stage whether individuals diagnosed with rhabdomyolysis are at high risk for kidney damage, allowing for earlier intervention in potential late complications during patient follow-up.

CPK is one of the basic biochemical markers widely used for diagnosis and monitoring in rhabdomyolysis cases [16]. Although this enzyme, released into the bloodstream as a result of muscle cell damage, plays a significant role in the diagnosis of rhabdomyolysis, it is not sufficient on its own to reflect the clinical course or predict the development of AKI. According to the literature, while some patients may have very high CPK levels, their renal functions may remain intact; in some cases, serious kidney damage may develop even at much lower CPK levels [32]. This variability demonstrates that making clinical decisions based solely on CPK levels is insufficient and points to the need for more comprehensive evaluation systems. In our study, similar to literature, we determined that creatine kinase level alone has a limited place in predicting the development of AKI. Different findings regarding the prognostic value of CPK clearly demonstrate the need for a holistic scoring system encompassing multidisciplinary parameters. However, it should be kept in mind that elevated CPK levels have been strongly linked to an increased risk of complications [33]. It has been emphasized in several studies that CPK levels above 5000 U/L are significantly associated with the development of AKI and the need for renal replacement therapy, while the overall risk of complications is also significantly increased in patients above this threshold [29]. Considering these findings, while regular monitoring of CPK levels remains important in the management of rhabdomyolysis, relying solely on CPK in clinical decisions is not sufficient. This underscores the need to develop holistic approaches that incorporate multiple parameters. GDF-15 is a biomarker that rises early in conditions such as cellular stress, inflammation, and tissue damage [34]. With this characteristic, it holds prognostic value and potential for clinical use in a wide range of systemic diseases, particularly cardiovascular and renal conditions [35]. However, there is no study in the literature regarding the use of GDF-15 for diagnostic or prognostic purposes specifically for rhabdomyolysis. There is a need for reliable biomarkers that can predict AKI due to rhabdomyolysis at an early stage. Our study has shown that GDF-15 could be a valuable biomarker in this context. The fact that GDF-15 may be elevated not only in rhabdomyolysis but also in various comorbidities such as cardiovascular diseases, malignancies, and systemic inflammatory conditions may limit its specificity [21]. Therefore, evaluating GDF-15 together with CPK, which is more specifically associated with rhabdomyolysis, increases diagnostic and prognostic accuracy; it provides stronger and more reliable results, especially in determining the risk of renal damage [36]. In recent years, a variety of biomarkers have been investigated to improve the early diagnosis and prognostic assessment of AKI. Among them, angiopoietin-2 (Ang-2) has gained particular interest due to its association with endothelial dysfunction and increased vascular permeability. Elevated serum levels of Ang-2 have been reported to correlate with the development of AKI in critically ill patients and have also been linked to poor outcomes in clinical conditions such as acute respiratory distress syndrome, acute pancreatitis, and myocardial infarction. As a marker of microvascular damage, Ang-2 may reflect early pathophysiological alterations that are not detectable by conventional renal function tests. In addition to Ang-2, other promising biomarkers have also been explored for their potential roles in the early identification and risk stratification of AKI [37,38,39,40]. Neutrophil gelatinase-associated lipocalin (NGAL) is released rapidly from renal tubular epithelial cells and shows a marked increase in both plasma and urine shortly after injury [41]. Systematic reviews in critically ill populations have demonstrated that NGAL can predict AKI well before serum creatinine begins to rise. Similarly, Kidney Injury Molecule-1 (KIM-1) is a highly specific indicator of proximal tubular damage and has been proposed as a reliable biomarker for both acute and chronic forms of kidney injury [42]. Taken together, evaluating Ang-2, NGAL, and KIM-1 in combination may improve risk stratification and provide further insights into the underlying mechanisms of AKI, particularly in cases related to rhabdomyolysis. However, their diagnostic and prognostic roles in rhabdomyolysis-associated AKI have not yet been systematically investigated.

In the scoring system developed by McMahon to assess the risk of AKI associated with rhabdomyolysis, several clinical and biochemical variables were utilized, including age, sex, presence of sepsis, serum calcium, phosphate, bicarbonate, creatinine, and CPK levels. However, the presence of sepsis and the need to assess numerous parameters limit the practical applicability of this scoring system in clinical settings [27]. In our study, sex distribution differed significantly among the groups (*p* = 0.046). While the control group demonstrated a balanced distribution between male (48.65%) and female (51.35%) participants, the proportion of males was notably higher in the trauma-induced rhabdomyolysis group (60.0%) and particularly elevated in the excessive exercise-induced rhabdomyolysis group (78.57%). This disparity is not attributed to sex-related differences in the clinical effects of rhabdomyolysis but rather to the higher incidence of trauma- and exertion-related rhabdomyolysis among males. The GDF-TRACK-AKI score has the potential to predict the development of AKI earlier and more specifically by considering a biomarker reflecting the cellular stress response, such as GDF-15, and clinically important factors such as the presence of trauma. Thus, it is thought that the GDF-TRACK-AKI score may provide a faster, more practical, and more sensitive risk classification, especially in high-risk patient groups such as trauma-related rhabdomyolysis.

Trauma stands out as both a factor that triggers pathophysiological processes in rhabdomyolysis and a risk factor that worsens the prognosis. In the literature, it has been shown that the risk of developing AKI is significantly higher, especially in cases of trauma-related rhabdomyolysis [43]. This increased risk is generally associated with factors such as widespread contusion of the muscle tissue caused by trauma, local and systemic inflammatory response, hypovolemia, reperfusion injury, and multi-organ involvement [12]. Therefore, in our study, additional scoring of the traumatic factor not only better reflected the clinical situation but also significantly increased the predictive power of the model for the development of AKI. Taking trauma into account makes an important contribution to early intervention and resource planning, especially in high-risk patient groups such as patients requiring intensive care monitoring [44].

The exclusion of certain rheumatologic and infectious diseases from the study was based on their potential to affect not only skeletal muscle but also renal function. These conditions may induce systemic inflammation and autoimmune responses, leading to muscle damage as well as direct or indirect impairment of kidney function. In such cases, elevated GDF-15 levels are likely to reflect the underlying systemic disease rather than rhabdomyolysis-specific pathology. Therefore, to prevent interpretive bias in biomarker-based analyses and to enhance the internal validity of the predictive model, these clinical scenarios were excluded. However, it should be acknowledged that this approach may artificially increase the sensitivity of the scoring system and limit its applicability to real-world clinical settings. Future studies should aim to include such complex patient groups to evaluate the generalizability and robustness of the proposed model across a broader clinical spectrum. Although such exclusions may enhance internal validity, they may also limit generalizability and lead to overestimation of predictive accuracy, which should be addressed in future broader cohort studies.

This research is subject to certain limitations. Primarily, the small sample size limits the extent to which the results can be generalized. Additionally, a balanced distribution of certain characteristics, such as sex, was not achieved among the rhabdomyolysis patients. Since GDF-15 levels may be influenced by the presence of other comorbidities, the results cannot be considered entirely independent of such effects. Furthermore, there may be unknown factors affecting GDF-15 levels that were not accounted for in this study. The single-center design limits the direct applicability of the findings to broader patient populations. However, given that the study was conducted at a tertiary referral hospital serving patients from diverse geographic regions, this limitation may be somewhat mitigated. Nonetheless, multicenter prospective studies with larger sample sizes are warranted to validate the accuracy and clinical utility of the developed scoring system. Moreover, since this study included only cases of trauma- and excessive exercise-induced rhabdomyolysis, the findings may not be generalizable to rhabdomyolysis arising from other etiologies. Future research focusing on different causes of rhabdomyolysis is recommended to broaden the applicability of these findings.

The findings of our study suggest that a simple but effective scoring system that can be used in clinical practice may be possible. The fact that the score can be calculated quickly and easily can accelerate clinical decision-making processes. It can also guide clinicians in evaluating the development of AKI in rhabdomyolysis patients.

## 5. Conclusions

AKI is widely recognized as both a prevalent and severe medical complication in individuals with rhabdomyolysis caused by trauma or excessive exercise. This research presented the GDF-TRACK-AKI score, an innovative system created to offer a reliable and user-friendly method for detecting the risk of acute kidney injury in the specified patient population. This approach integrates circulating concentrations of GDF-15 and creatine kinase with cases of rhabdomyolysis caused by trauma. While creatine kinase is commonly used to diagnose rhabdomyolysis, relying solely on it may not adequately forecast the onset of acute kidney injury. Including GDF-15, a marker of initial cellular inflammation and stress, greatly enhances the precision of predictions and practical application in clinical settings. Assessing GDF-15 together with creatine kinase provides a more robust approach for early identification of acute kidney injury risk in patients suffering from trauma- or exercise-related rhabdomyolysis. In conclusion, the GDF-TRACK-AKI score acts as a reliable and effective tool for the early identification of acute kidney injury in cases of rhabdomyolysis. However, broader use in clinical practice demands additional validation across large and diverse groups of patients.

## Figures and Tables

**Table 1:** Child–Pugh classification. **Definition:** A scoring system used to evaluate the severity of chronic liver disease.

Parameter	1 Point	2 Points	3 Points
Bilirubin (mg/dL)	<2	2–3	>3
Albumin (g/dL)	>3.5	2.8–3.5	<2.8
INR	<1.7	1.7–2.3	>2.3
Ascites	None	Mild	Moderate to Severe
Hepatic Encephalopathy	None	Grade I–II	Grade III–IV

Grades: A (5–6 points), mild B (7–9 points), moderate C (10–15 points).

**Table 2:** NYHA classification for heart failure. Definition: A classification system for heart failure based on the patient’s tolerance to physical activity.

Class	Description
I	No limitation of physical activity. Ordinary activities do not cause symptoms.
II	Slight limitation of physical activity. Ordinary activity results in symptoms.
III	Marked limitation. Symptoms occur with less than ordinary activity.
IV	Symptoms are present even at rest. Any physical activity leads to discomfort.

**Table 3:** GOLD classification for COPD. Definition: A system that classifies the severity of chronic obstructive pulmonary disease (COPD) based on symptoms and spirometry findings.

Stage	FEV₁ (% Predicted)	Description
GOLD 1	≥80%	Mild COPD
GOLD 2	50–79%	Moderate COPD
GOLD 3	30–49%	Severe COPD
GOLD 4	<30%	Very Severe COPD

**Table 4:** Demographic and clinical characteristics of study groups.

Variable	Category	Group						*p*
		Rhabdomyolysis After Trauma		Control		Rhabdomyolysis After Excessive Exercise		
		n	%	n	%	n	%	
**Sex**	Female	12	40.00	19	51.35	6	21.43	0.046 *
	Male	18	60.00	18	48.65	22	78.57	
Renal Replacement Therapy	Absent	5	16.67	37	100.00	28	100.00	0.001 *
	Present	25	83.33	0	0.00	0	0.00	
Mortality	Absent	29	96.67	37	100.00	28	100.00	0.335
	Present	1	3.33	0	0.00	0	0.00	
Acute Kidney Injury	Absent	5	16.67	37	100.00	26	92.86	0.001 *
	Present	25	83.33	0	0.00	2	7.14	
**Variable**		**Median ± IQR**	**Min.-** **Maks.**	**Median ± IQR**	**Min.** **-Maks.**	**Median ± IQR**	**Min.-** **Maks.**	***p***
Age		33.00 ± 14.25	18 85	75.00 ± 19.00	22 87	22.50 ± 14.25	18 65	0.001 *
GDF-15 Level (pg/mL)		5741.50 ± 2830.50	2200 7494	2378.00 ± 1314.00	1211 4722	2958.50 ± 1386.00	1467 5056	0.001 *

Statistical differences among the groups were evaluated using the Chi-square test for categorical variables and the Kruskal–Wallis test for continuous variables due to the non-normal distribution of the data. A *p*-value less than 0.05 was considered statistically significant and indicated with an asterisk (*).

**Table 5:** Correlation between serum GDF-15 levels and laboratory parameters.

Variable	Correlation Coefficient (r)	*p*-Value
Creatine Kinase (U/L)	0.517	0.001 *
Creatinine (mg/dL)	0.517	0.001 *
Urea (mg/dL)	0.649	0.001 *
Glomerular Filtration Rate (mL/min/1.73 m^2^)	−0.568	0.001 *
Uric Acid (mg/dL)	−0.026	0.799
Aspartate Aminotransferase (U/L)	−0.01	0.927
Alanine Aminotransferase (U/L)	0.082	0.43
Alkaline Phosphatase (U/L)	0.281	0.006 *
Gamma-Glutamyl Transferase (U/L)	0.057	0.582
Lactate Dehydrogenase (U/L)	0.175	0.09
Total Bilirubin (mg/dL)	0.27	0.008 *
Direct Bilirubin (mg/dL)	−0.043	0.682
Sodium (mmol/L)	−0.409	0.001 *
Potassium (mmol/L)	0.228	0.026 *
Calcium (mg/dL)	0.207	0.044
Hemoglobin (g/dL)	0.008	0.804
Platelet (×10^3^/µL)	−0.025	0.81
White Blood Cell Count (×10^3^/µL)	0.597	0.001 *

Spearman’s correlation analysis was used. Statistically significant correlations (*p* < 0.05) are marked with an asterisk (*).

**Table 6:** Comparison of serum GDF-15 levels according to clinical variables.

Category	Subgroup	GDF-15 Median	IQR	Test Value (t)	*p*-Value
Sex	Female	3267.00	2222.00	−0.530	0.596
	Male	2991.50	2471.00		
Renal Replacement Therapy	Absent	2639.00	1276.75	−6.233	0.001 *
	Present	5794.00	2125.50		
Acute Kidney Disease	Absent	2608.50	1251.75	−6.514	0.001 *
	Present	5756.00	2006.00		
Rhabdomyolysis Etiology	Trauma	5741.50	2830.50	−4.808	0.001 *
	Excessive exercise	2958.50	1386.00		
Group Comparison (Rhabdomyolysis vs. Control)	Rhabdomyolysis	3580.50	3108.50	−4.759	0.001 *
	Control	2378.00	1314.00		

Group comparisons were performed using the Mann–Whitney U test. Statistically significant differences (*p* < 0.05) are indicated with an asterisk (*). Serum GDF-15 levels are presented as median and interquartile range (IQR).

**Table 7:** Laboratory findings by acute kidney injury status in trauma or excessive exercise induced rhabdomyolysis patients.

Variable	Acute Kidney Injury				Mann–Whitney U Test	
	Absent		Present			
	Median	IQR	Median	IQR	z	*p*
GDF-TRACK-AKI score	0.00	0.00	3.00	2.00	−8.079	0.001 *
Creatine Kinase (U/L)	174.00	13,966.75	33,976.00	79,574.00	−5.875	0.001 *
Creatinine (mg/dL)	0.84	0.25	5.29	2.92	−7.576	0.001 *
Urea (mg/dL)	28.00	15.75	92.00	65.00	−5.434	0.001 *
Glomerular Filtration Rate (mL/min/1.73 m^2^)	101.50	38.75	20.00	43.00	−5.496	0.001 *
Uric Acid (mg/dL)	4.70	1.98	5.50	3.40	−1.250	0.211
Aspartate Aminotransferase (U/L)	39.00	9.00	24.00	8.00	−6.296	0.001 *
Alanine Aminotransferase (U/L)	39.00	93.00	68.00	60.00	−2.009	0.044 *
Alkaline Phosphatase (U/L)	36.50	76.75	67.00	64.00	−1.787	0.074
Gamma-Glutamyl Transferase (U/L)	26.00	40.00	48.00	59.00	−1.585	0.113
Lactate Dehydrogenase (U/L)	260.50	197.00	480.00	326.00	−2.695	0.007 *
Total Bilirubin (mg/dL)	0.50	0.38	0.60	0.90	−4.464	0.001 *
Direct Bilirubin (mg/dL)	0.20	0.20	0.20	0.40	−0.896	0.370
Sodium (mmol/L)	139.00	3.75	135.00	9.00	−1.770	0.077
Potassium (mmol/L)	4.25	0.050	59.382	0.90	−3.445	0.001 *
Calcium (mg/dL)	9.00	0.70	8.50	0.50	−0.116	0.908
Hemoglobin (g/dL)	12.65	3.67	8.80	1.70	−3.854	0.001 *
Platelet (×10^3^/µL)	266.50	151.25	249.00	195.00	−4.869	0.001 *
White Blood Cell Count (×10^3^/µL)	8.13	3.80	13.77	5.56	−0.153	0.879

Comparisons between patients with trauma or excessive exercise-induced rhabdomyolysis, stratified by AKI status, were performed using the Mann–Whitney U test. Data are presented as median and interquartile range (IQR). Statistically significant differences (*p* < 0.05) are marked with an asterisk (*).

**Table 8:** Distribution of GDF-Track AKI scores according to AKI status.

Variable	Acute Kidney Injury				Mann–Whitney U Test	
	Absent		Present			
	Median	IQR	Median	IQR	t	*p*
GDF Track AKI score	0.00	0.00	3.00	2.00	−8.079	0.001 *

Comparison of GDF-TRACK-AKI scores between patients with and without acute kidney injury (AKI) was conducted using the Mann–Whitney U test. Data are presented as median and interquartile range (IQR). A *p*-value < 0.05 was considered statistically significant and is indicated with an asterisk (*).

**Table 9:** Diagnostic performance metrics of GDF-TRACK-AKI score for predicting acute kidney injury.

Variable	Cut-off Value	AUC (95% CI)	Sensitivity (%)	Specificity (%)	PPV (%)	NPV (%)	Youden Index
Acute Kidney Injury	2.5	0.918 (0.857–0.979)	67.0%	98.0%	94.4%	81.4%	0.65

The ROC curve demonstrated excellent diagnostic performance, with an AUC of 0.918 (95% CI: 0.857–0.979) for predicting AKI. Abbreviations: AUC, area under the curve; CI, confidence interval; PPV, positive predictive value; NPV, negative predictive value.

## Data Availability

The original contributions presented in this study are included in the article. Further inquiries can be directed to the corresponding authors.

## Abbreviations

AKI	Acute Kidney Injury
ALT	Alanine Aminotransferase
ANOVA	Analysis of Variance
AST	Aspartate Aminotransferase
ATP	Adenosine Triphosphate
AUC	Area Under the Curve
CI	Confidence Interval
CK	Creatine Kinase
COPD	Chronic Obstructive Pulmonary Disease
CPK	Creatine Phosphokinase
ELISA	Enzyme-Linked Immunosorbent Assay
GDF-15	Growth Differentiation Factor-15
GDF-TRACK-AKI	Growth Differentiation Factor-15, Trauma, Creatine Kinase Acute Kidney Injury Score
GFR	Glomerular Filtration Rate
GGT	Gamma-Glutamyl Transferase
GOLD	Global Initiative for Chronic Obstructive Lung Disease
HIV	Human Immunodeficiency Virus
KDIGO	Kidney Disease Improving Global Outcomes
LDH	Lactate Dehydrogenase
NPV	Negative Predictive Value
NYHA	New York Heart Association
OR	Odds Ratio
PPV	Positive Predictive Value
ROC	Receiver Operating Characteristic
RRT	Renal Replacement Therapy
SD	Standard Deviation
SPSS	Statistical Package for the Social Sciences
WBC	White Blood Cell
